# The Pitch Imagery Arrow Task: Effects of Musical Training, Vividness, and Mental Control

**DOI:** 10.1371/journal.pone.0121809

**Published:** 2015-03-25

**Authors:** Rebecca W. Gelding, William Forde Thompson, Blake W. Johnson

**Affiliations:** 1 Department of Cognitive Science, Macquarie University, Sydney, Australia; 2 ARC Centre of Excellence in Cognition and its Disorders, Macquarie University, Sydney, Australia; 3 Department of Psychology, Macquarie University, Sydney, Australia; The University of Chicago, UNITED STATES

## Abstract

Musical imagery is a relatively unexplored area, partly because of deficiencies in existing experimental paradigms, which are often difficult, unreliable, or do not provide objective measures of performance. Here we describe a novel protocol, the Pitch Imagery Arrow Task (PIAT), which induces and trains pitch imagery in both musicians and non-musicians. Given a tonal context and an initial pitch sequence, arrows are displayed to elicit a scale-step sequence of imagined pitches, and participants indicate whether the final imagined tone matches an audible probe. It is a staircase design that accommodates individual differences in musical experience and imagery ability. This new protocol was used to investigate the roles that musical expertise, self-reported auditory vividness and mental control play in imagery performance. Performance on the task was significantly better for participants who employed a musical imagery strategy compared to participants who used an alternative cognitive strategy and positively correlated with scores on the Control subscale from the Bucknell Auditory Imagery Scale (BAIS). Multiple regression analysis revealed that Imagery performance accuracy was best predicted by a combination of strategy use and scores on the Vividness subscale of BAIS. These results confirm that competent performance on the PIAT requires active musical imagery and is very difficult to achieve using alternative cognitive strategies. Auditory vividness and mental control were more important than musical experience in the ability to perform manipulation of pitch imagery.

## Introduction

Musical imagery can be described as “hearing a tune in your head” [1]. It is a common, everyday experience even for those with no musical training. Despite the ubiquity of musical imagery, research on the topic has often examined musicians and non-musicians separately. This is largely because of deficiencies in existing experimental protocols for inducing and measuring musical imagery. In some cases, imagery tasks are too difficult for non-musicians to complete [2]; in other cases, they are too easy for musicians and susceptible to ceiling effects for this population [3]. Other studies have considered musicians with a range of abilities and correlated their performance on imagery tasks with years of musical training [4], or cumulative hours of training [5]. In general, musicians perform better than non-musicians on musical imagery tasks [2, 6]. However, it is not always clear how to interpret such findings because musical knowledge is sometimes needed in order to understand and/or complete these tasks.

Most studies of musical imagery require participants to maintain an image of familiar melodies or scales [7]. These tasks require a variety of judgments including: pitch judgments about two syllables of the lyrics associated with an imagined tune [8]; comparing the similarity of two imagined instrumental timbres [9]; imagining the continuation of ascending musical scales [10, 11]; imagining familiar music during gaps in the presentation [12, 13]; imagining the continuation of a familiar melody and judging an audible tone for accuracy, as the last tone of the melody [14].

Other experimental paradigms are designed to induce dynamic musical imagery, and hence require participants to shift from one musical image to another through effortful manipulation. These paradigms have included tasks that require mentally transposing a melody into a different key or imaging the pitches of a melody in reverse (retrograde) order [2, 5].

This distinction—between maintenance and manipulation of mental imagery—has also been the subject of investigation in other modalities, particularly in visual imagery. Studies have shown that the ability to form vivid visual images is psychometrically distinct from the ability to manipulate those images in space. In one study, visual artists were found to excel at maintaining object imagery but had difficulty with spatial manipulation of images, whereas engineers and scientists exhibited the opposite pattern of performance [15]. In the auditory domain, Hansen et al. found that musicians could recall longer sequences of spoken digits than non-musicians, but they were no better than non-musicians at a backward digit span task that required mental manipulation of that verbal image [16]. These findings suggest that tasks requiring a large store of musical sequences in short-term memory, such as those required in maintenance paradigms, are biased towards musicians. As such, tasks requiring the mental manipulation of musical materials with minimal burden on short-term memory may be better suited to study the role of training on musical imagery.

Another area of individual differences that has received relatively little attention is the vividness of auditory imagery among nonclinical populations [17]. The Bucknell Auditory Imagery Scale (BAIS) is a self-report questionnaire that incorporates a *vividness* subscale (BAIS-V) and a *control* subscale (BAIS-C), with the former measuring the subjective clarity of an image and the latter measuring the ease with which participants can change or manipulate an auditory image at will [18]. Previous studies have shown that results from the BAIS, particularly BAIS-V, correlate with performance on musical imagery tasks [2, 19, 20]. The BAIS-C has also been shown to correlate significantly with performance on a pitch discrimination task where participants were required to indicate which of two tones was higher in pitch [20]. Interestingly, BAIS scores have repeatedly been shown to correlate only mildly (*r* ~.30) with musical training or experience [19, 20].

This investigation employed a novel protocol for evaluating musical imagery—the *Pitch Imagery Arrow Task* (PIAT). Several considerations informed the design of this task. First, a number of paradigms that purport to measure musical imagery do not provide any objective behavioral confirmation that participants have actually used musical imagery [1, 6]. For example, Kraemer et al. [12] had participants passively listen to familiar and unfamiliar music that had silent pauses of 2–5 s inserted. They then examined brain activity during the period of silence. Although subjects were not instructed to imagine the tunes, all participants reported musical imagery during gaps in the familiar music but not during gaps in the unfamiliar music. Yoo et al. [21] had participants familiarise themselves with a single pitch outside of the scanner and then were required to imagine the same pitch while their brain was scanned using fMRI. In these studies, imagery was assumed or argued to have occurred either because participants were explicitly instructed to form images or because the investigators created a context in which imagery was likely to occur [1, 6].

Second, although some imagery tasks have an objective behavioural outcome measure, they are so simple or repetitive that musical imagery may be unnecessary to perform the task. For example, Janata and Paroo used familiar ascending scales in a number of their studies, in order to “force listeners to move their mental images in pitch space” [10]. However, these authors acknowledged that by using familiar scales and confirming their use of imagery only for the last note in the sequence (i.e. the tonic), participants may have used the initial (tonic) note to infer the final note of the scale (tonic one octave above the initial scale note). That is, they were not obliged to imagine each note of the scale [10]. Herholtz et al. [14] required participants to listen to one of nine familiar nursery rhyme introductions (16 repetitions per melody), then imagine the series of missing tones and were tested on the last note of the sequence. This repeated exposure may have led to a learned association between the start of the sequences and the sounded last note, rather than the use of musical imagery to continue the missing tones. To combat this they asked participants whether they had used “any other strategy than imagining the melody, in order to fulfil the task of judging the correctness of the test tone” [14]. Having participants confirm the type of strategy used to complete a given imagery task can be an additional way of ensuring auditory imagery is used rather than an alternative cognitive strategy [22], though such a question should be worded in a way that does not bias participants in their response.

At the other extreme are protocols that are so difficult that only expert musicians can perform them, such as the mental reversal of familiar melodies [2]; see also [5, 7]. As only highly trained participants can complete this task, this limits the range and generalizability of the results.

Finally it has been said that the study of imagery stands precisely at the intersection of two key branches of cognitive psychology—perception and memory [23]. As such, we have included both a Perception control condition and a Mental Arithmetic control condition to be able to compare imagery performance (accuracy and reaction time) with both musical perception and non-musical working memory.

The PIAT has a number of advantages over existing protocols for evaluating imagery. Specifically, the task (1) requires a behavioural response to objectively measure accuracy and response times of imagery performance; (2) is extremely difficult to successfully perform using alternative cognitive strategies other than pitch imagery; (3) employs novel rather than familiar sequences of pitches that cannot be anticipated in advance; (4) employs a range of difficulties implemented in a staircase design, such that it can induce imagery in participants with a wide range of musical experience; (5) incorporates a pitch perception control condition, permitting the assessment of differences in strategies and brain mechanism for imagery versus perception; (6) incorporates a mental arithmetic control condition, permitting the assessment of differences in strategies and brain mechanisms for imagery tasks that employ different cognitive computations.

Our primary goal was to verify the efficacy of the PIAT in inducing and training pitch imagery in musicians and non-musicians with a wide range of musical experience. We also investigated the role of musical training, imagery vividness, and mental control in predicting performance. We hypothesised that (1) participants who used a pitch imagery strategy would show significantly better performance than participants who employed alternative cognitive strategies; (2) successful task performance should be determined more by an individual’s vividness and control of musical images (as indexed by the BAIS), than one’s history of musical training.

## Methods

### Participants

24 trained musicians and 16 non-musicians were recruited for the study. All participants self-reported to being right-handed, having normal or corrected-to-normal vision and normal hearing. Only right-handed individuals were recruited. Participants were classified as musicians if they had more than 5 years of continuous formal music lessons and have been actively playing their instrument in the last 2 years. Non-musicians were defined as those with less than 2 years formal training, or those who had been non-active in their instrument for more than 10 years. All participants were classified as either a musician or a non-musician based on these criteria. All participants provided written consent and all procedures were approved by the Macquarie University Human Research Ethics Committee. Table 1 summarises the characteristics of the two groups. The musicians and non-musicians did not differ significantly in age, gender, daily hours spent listening to music, or education, but they did differ significantly in the Musical Experience Index (MEI). This was calculated as the number of years spent actively playing an instrument / singing, either through formal lessons or self-taught, divided by current age to obtain a percentage of musical experience over the lifetime. For example, if a participant had piano lessons for 2 years, then stopped playing, and later taught themselves to play guitar for 3 years, and are currently aged 25, their musical experience index was (2 + 3)/25 = 0.2. However if these lessons had happened concurrently then the total years of playing music would be 3, and so their MEI would be 3/25 = .12. This index was used to normalise the musical training across the wide age range of participants. The musicians on average had spent 45% of their life’s years actively participating in music, while non-musicians had spent on average 12%, as seen in Table 1.

**Table 1:** Summary of the demographic details of the participants.

	Mean Age (Range)	Number of Females	Mean Musical Experience Index—MEI (range)	Mean Daily Hours spent listening to music(range)
Musician (N = 24)	26.2 (18–48)	15	.45 (.16-.72)	2.5 (0.12–10)
Non-Musician (N = 16)	22.6 (18–41)	7	.12 (0-.28)	1.5 (0.25–4)
Total Sample (N = 40)	24.7 (18–48)	22	.34 (0-.72)	2.1(0.12–10)

### Stimuli

#### Pitch Imagery Condition

**Fig 1:**
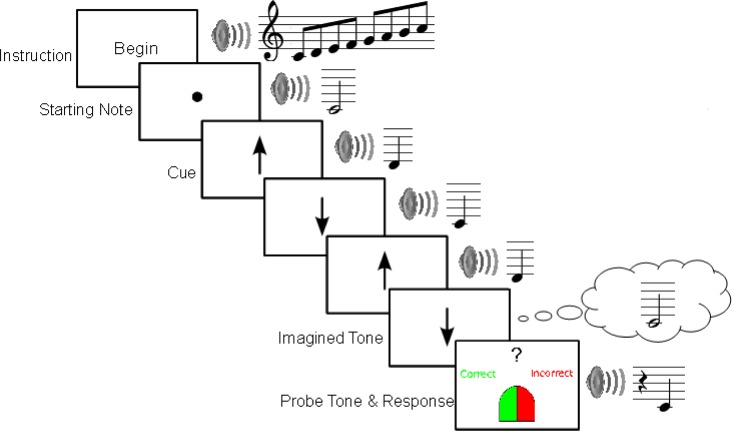
Schematic of the Pitch Imagery Arrow Task (PIAT).

The PIAT, as outlined in Fig. 1, consists of three successive components: (1) a *setup* component in which the participant listens to a starting sequence of computer-generated piano notes. Each successive note is immediately up or down the major scale relative to the preceding note, with the scale direction of the next note (ascending or descending) being random and unpredictable from note to note. (2) An *imagery* (continuation) component in which the piano notes stop while the participant is prompted to imagine a series of 1–5 notes continuing from the last heard note, prompted by vertical up or down arrows which indicate the scale direction for each succeeding note to be imagined; (3) A *probe* component which presents a heard piano note that either matches or does not match the last of the notes in the imagery sequence. After hearing the probe participants were required to indicate with a button press if the probe is a match or a mismatch to the last note of the imagined sequence.

The setup component of each trial began with an ascending major scale that started and ended on the tonic, to provide participants with a tonal context that they could use as a frame of reference for their subsequent judgments [24]. Following the key-defining scale, the starting note of the test sequence is played for a duration of 2 s, and a fixation circle appears in the middle of the computer screen. Following the starting note, each successive note was randomly selected from a position immediately up or down the scale from the last note at a rate of one per second, and played for a duration of 500 ms. Each note was accompanied on the visual display with an up or down arrow that indicated the scale direction from the preceding note.

In the imagery component, one to five arrows were then presented in silence at the same rate as the preceding note / arrow combinations of the setup phase and participants were required to imagine the next scale note up or down from the previous (heard or imagined) note as indicated by the scale direction arrows.

In the probe component of each trial, a target screen with an image of a mouse indicating left click for “Correct” and right click for “Incorrect” was displayed for 1 second to alert participants to an impending probe. A probe tone was then played and participants were required to indicate whether the probe tone matched or did not match the final note of the imagery component. If no response was recorded within 4 seconds the trial was coded as a missed trial, and participants received a warning message to respond more quickly on future trials. Feedback was provided on each trial to advise the participant whether their response was accurate or not.

The PIAT was programmed with five levels of imagery difficulty, corresponding to the number of sequential notes (from 1–5) in the imagery component. Each imagery level contained three stages that manipulated the complexity of the setup component, in terms of the maximum number of audible notes in the setup sequence, the key signature for the sequence, and whether the starting note was a tonic or dominant. In particular, in stages 2 and 3 the key signatures were randomised from a possible 5 key signatures (C Major, C# Major, D Major, Eb Major or E Major). These were set so as not to repeat the previous trial to ensure variability in each stage [10]. Table 2 summarizes the attributes for each level and stage. The number of sequential notes in the setup component was also randomised between trials so that participants were unable to predict when they would be required to begin the imagery component.

**Table 2:** Summary of Level and Stage Design of the Imagery and Mental Arithmetic (MA) Conditions.

Level	Stage	Key Signature	Starting Note	No. of arrow with heard tones	No. of imagined tones (Imagery)	Starting Number for MA	No. of arrows for MA	Numeral presented with arrow for MA
1	1	C	tonic	3	1	1	4	1
	2	*C, C#, D, Eb, E	tonic	*3–4		*1–5	*4–5	
	3	*C, C#, D, Eb, E	dominant	*3–6		*1–5	*4–7	
2	1	C	tonic	*3–4	2	1	*5–6	*1–2
	2	*C, C#, D, Eb, E	tonic	*3–5		*1–5	*5–7	
	3	*C, C#, D, Eb, E	dominant	*3–6		*1–5	*5–8	
3	1	C	tonic	*3–4	3	1	*6–7	*1–3
	2	*C, C#, D, Eb, E	tonic	*3–5		*1–5	*6–8	
	3	*C, C#, D, Eb, E	dominant	*3–6		*1–5	*6–9	
4	1	C	tonic	*3–4	4	1	*7–8	*1–4
	2	*C, C#, D, Eb, E	tonic	*3–5		*1–5	*7–9	
	3	*C, C#, D, Eb, E	dominant	*3–6		*1–5	*7–10	
5	1	*C, C#, D, Eb, E	tonic	*3–4	5	1	*8–9	*1–5
	2	*C, C#, D, Eb, E	dominant	*3–5		*1–5	*8–10	
	3	*C, C#, D, Eb, E	*tonic or dominant	*3–6		*1–5	*8–11	

The Perception Conditions corresponded to the first five columns of the table.

* denotes when a random variable from those listed could be used at that stage. Only the key signature was set so as not to repeat for a concurrent trial.

The probe tone was a match for 50% of the trials. For mismatch trials the probe tone was always in the same key and within 2 steps of the correct answer. The maximum possible range of notes for each trial was set to 3 scale steps up or down from the starting note. For example, for a trial in C Major, beginning on Middle C (C4), the tones (both heard or to be imagined) were within the range of G below middle C (G3) and F above middle C (F4).

#### Pitch Perception Condition

The Pitch Perception trials were identical to the imagery trials, except that arrows were always paired with heard notes during the continuation component. In this case the last note in the sequence then became the correct response for the probe.

#### Mental Arithmetic Condition

The Mental Arithmetic trial’s start screen showed “Begin Mental Arithmetic”, then a number appeared on the screen which was the starting point of the sequence. The up and down arrows also included a number at their point which indicated how much to increase (up) or decrease (down) the running total by. After a random number of arrows were presented, which varied from a minimum of 4 (Level 1, Stage 1) to a maximum of 11 (Level 5, Stage 3), the same target screen with the mouse image then appeared. After 1 second, a number appeared at the top of the screen indicating the probe number. Participants then responded in the same way as the musical trials to confirm if the number was correct or incorrect. Incorrect answers were presented on 50% of the trials and were always 1 integer away from the correct number. The mental arithmetic trials also increased in difficulty as the levels progressed, as per Table 2, however the sequences were set to remain in a range of 0–10. This range was determined after pilot testing as participants reported being unable to successfully and consistently compute larger numbers at the required rate of one arrow per second (as per the music trials).

#### Bucknell Auditory Imagery Scale

In order to assess the role of self-reported auditory vividness and control on pitch imagery performance, the participants also completed the Bucknell Auditory Imagery Scale (BAIS) [18]. The scale includes two sub scales, for vividness (BAIS-V) and control (BAIS-C), both of which have 14 items each. BAIS-V questions required participants to rate how clearly they could imagine a particular auditory image from 1–7, such as a trumpet beginning to play “Happy Birthday”, with 1 indicating that no image was present at all, 4 being fairly vivid and 7 being as vivid as actual sound. BAIS-C questions required participants to rate similarly from 1–7, how easily they could then change an image from, for example, the trumpet beginning the piece to a violin continuing the song. Previous studies have shown that results from the BAIS-V, correlate with performance on musical imagery tasks [2, 19, 20], but no previous studies have reported a correlation with BAIS-C and imagery performance.

### Procedure

Presentation software (www.neurobs.com) was used to control the experiment and to record responses. Acoustic stimuli were generated from the 'Piano' instrument sound by Finale 2012 software (Makemusic Inc; Eden Prairie, MN) and exported as .wav files for use in Presentation.

Upon being seated in front of the computer, participants were given a sound check, whereby they could manually adjust the volume of the tones to a suitable level. They were then introduced to the three types of trials, and given a simple example of a Pitch Imagery trial and a Mental Arithmetic trial. Participants were informed that no movement or humming was allowed to assist them with the task, but they should “as vividly as possible, imagine the tones and keep their bodies still”. An opportunity for questions was given prior to the start of the task. There were 90 Pitch Imagery Trials, 30 Pitch Perception Trials, and an average of 22 Mental Arithmetic Trials (range 14–40). The Pitch Perception trials were randomly interleaved with the Imagery trials after an initial 10 Imagery trials were presented. The accuracy of response for the Perception trials did not impact on the progression of the participant through the task. Mental Arithmetic trials were presented as participants moved between stages; one trial if moving up a stage or level, and two trials if moving down a level. The average time taken to complete the task was 53 mins.

The task also included a fast exit in which participants who failed to successfully progress through Level 1 of the Imagery Trials on more than 3 attempts (that is, got more than 18 incorrect responses for Level 1 Imagery Trials) were excused from further trials. These participants were deemed to have found the task too difficult or failed to understand how to complete it. At each point of failing Level 1, the participants were given the opportunity to ask questions and the requirements of the task were reiterated verbally.

Upon completion, participants were visually presented with their percent correct scores for each Imagery level, as well as overall percent correct for the Perception and Mental Arithmetic conditions. They were then asked verbally to rate from 1–5 overall how vividly or clearly they formed the musical images during the task (1—not at all vivid; 5—very vivid) [14, 25]. They were also asked: “What strategies did you use to complete the musical imagery task?” Responses were written down and later categorised into one of several groups during analysis. Participants then completed a musical experience questionnaire which included questions of past and current, both formal and informal musical participation, as well as the BAIS.

## Results

Four participants failed to progress past Level 1 and were excluded from the final analyses. All four excluded participants were non-musicians with an average Musical Experience Index of .04, that is had actively participated in playing music for 4% of their lives (range 0–.12).

### Overall Accuracy and Reaction Times

A 2 x 3 ANOVA of Accuracy (Group: Musician, Non-Musician) x Condition (Imagery, Perception, Mental Arithmetic) revealed a significant main effect for Condition, *F*(2,102) = 4.46, *p* = .01, η^2^ = .07 but no significant main effect for Group *F*(1,102) = 0.04, *p* = .83. The Group x Condition interaction was also significant *F*(2,102) = 6.58, *p* = .002, η^2^ = .10, due to the fact that musicians were more accurate than non-musicians on the Imagery (musicians: M = .820, SD = 0.09; non-musicians: M = .763, SD = 0.05; *t*(34) = 2.07, *p* = .046, *d* = 0.75) and Perception conditions (musicians: M = .906, SD = 0.11; non-musicians: M = .833, SD = 0.14; *t*(34) = 1.714, *p* = .096, *d* = 0.61), while non-musicians were more accurate than musicians in the Mental Arithmetic condition (musicians: M = .795, SD = 0.16; non-musicians: M = .910, SD = 0.06; *t*(34) = 2.351, *p* = .025, *d* = 0.86).

A 2 x 3 ANOVA of Mean Hit Reaction Times (Group (Musician / Non-Musician) x Condition (Imagery; Perception; Mental Arithmetic) revealed a significant main effect of Group (*F*(1,102) = 6.167, *p* = .02, η^2^ = .05), with musicians showing slower overall reaction times than non-musicians. There was also a significant main effect of Condition (*F*(2,102) = 5.034, *p* = .008, η^2^ = .08). Post hoc paired t tests showed that reaction times (ms) were not significantly different for the Imagery (M = 1027.9, SD = 215.6) and Perception conditions (M = 992.3, SD = 250.6), but differed significantly between Imagery and Mental Arithmetic (M = 845.1, SD = 317.6): (*t*(35) = 4.92, p < .001, *d* = 0.82), as well as Perception and Mental Arithmetic: (*t*(35) = 3.38, *p* = .002, *d* = 0.56). All post hoc tests used the Bonferroni correction procedure with a critical alpha of .05/3.

**Fig 2:**
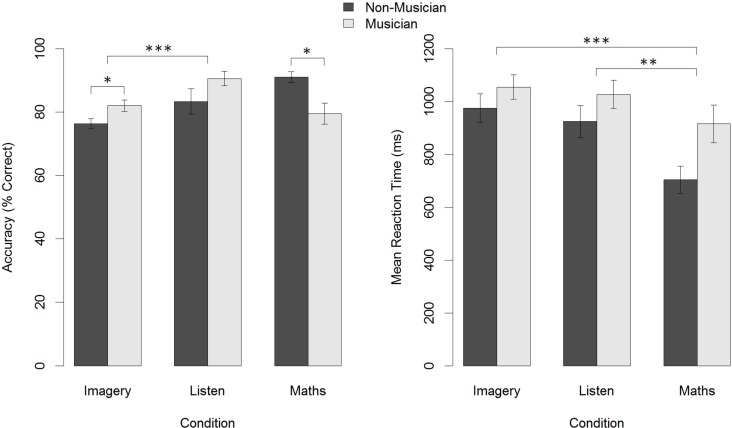
Accuracy and reaction time on the PIAT. * = *p* < .05, ** = *p* < .01, *** = *p* < .001.

There was no significant interaction Group x RT interaction (*F*(2,102) = 0.589, *p* = .56, η^2^ = .01). Fig. 2 shows a summary of these results.

### Bucknell Auditory Imagery Scale

BAIS-V and BAIS-C were correlated across the three conditions for overall accuracy (percent correct) and mean hit reaction times. BAIS-V was significantly correlated with Imagery accuracy (*r* = .49, *p* = .002). BAIS-C was significantly correlated with overall accuracy for the Imagery (*r* = .59, *p <* .001) and the Perception condition (*r* = .33, *p* = .049) as well as significantly negatively correlated with the mean hit reaction times for both the Imagery condition (*r* = -0.51, *p* = .001) and the Perception condition (*r* = -0.39, *p* = .019).

The BAIS measures were also correlated significantly with a number of other variables. The participant’s debriefing vividness rating was significantly correlated with BAIS-V (*r* = .51, *p* = .001), though not with BAIS-C (*r* = .25, *p* = .15).

The MEI showed a significant positive correlation with BAIS-C (*r* = .41, *p* = .014), but not with BAIS-V (*r* = .24, *p* = .17). However an independent t-test revealed musicians scored significantly higher than non-musicians only on BAIS-V; (musicians: M = 5.40, SD = 0.77; non-musicians: M = 4.71, SD = 1.13; *t*(34) = 2.16, *p* = .038, *d* = 0.76), not BAIS-C (musicians: M = 5.53, SD = 0.91; non-musicians: M = 5.03, SD = 0.88; *t*(34) = 1.57, *p* = .125, *d* = 0.55). Table 3 shows the correlation matrix for all variables of interest.

**Table 3:** Correlation Matrix of key variables.

	% Correct Imagery	% Correct Perception	% Correct Maths	Ave RT Imagery	Ave RT Perception	Ave RT Maths	BAIS Vividness	BAIS Control	Debrief Vividness	Musician / Non-Musician	Musical Experience Index	Musical Imagery Strategy Used [Y = 1;N = 0]	Maximum Level
% Correct Imagery	1.000	0.488* *	0.075	−0.479*	−0.409	−0.337**	0.555**	0.674* **	0.497** *	0.315*	0.483* *	0.614* **	0.791* **
% Correct Perception		1.000	0.134	−0.219	−0.478*	−0.171	0.271	0.353*	0.358*	0.268	0.370*	0.604* **	0.519* **
% Correct Maths			1.000	−0.253	−0.252	−0.674** *	−0.240	−0.088	−0.133	−0.40*	−0.171	0.108	−0.030
Ave RT Imagery				1.000	0.672* **	0.735** *	−0.203	−0.51**	−0.234	0.165	−0.152	−0.313	−0.382*
Ave RT Perception					1.000	0.639** *	−0.064	−0.380*	−0.197	0.175	−0.194	−0.415*	−0.300
Ave RT Maths						1.000	0.046	−0.309	−0.073	0.335	−0.025	−0.255	−0.161
BAIS Vividness							1.000	0.713* **	0.546**	0.357*	0.237	0.270	0.484* *
BAIS Control								1.000	0.281	0.275	0.408*	0.496* *	0.475* *
Debrief Vividness									1.000	0.331*	0.307	0.517* *	0.576* **
Musician / Non-Musician										1.000	0.710* **	0.270	0.323*
Musical Experience Index											1.000	0.549* **	0.458* *
Musical Imagery Strategy Used [Y = 1;N = 0]												1.000	0.650* **
Maximum Level													1.000

Significance is denoted as

* = *p* < .05

** = *p* < .01

*** = *p* < .001.

### Strategy Use

An open-ended question asked participants to describe the strategies they had used for performing the imagery task. Responses fell into two broad categories, *Musical Imagery*, or *Alternative Strategy*. Musical imagery strategy users (N = 21) reported hearing the sound in their head or singing the notes in their head thereby following the arrows and hearing the sounds in their minds throughout the imagery (continuation) component of the task. Alternative Strategy users (N = 14) reported a variety of alternative cognitive strategies such as counting arrows, using intuition or visual imagery. These participants were keeping track of the movement of the arrows, but using musical imagery only at the end of the trial, to make a judgement regarding whether the test tone was correct. As the goal of the PIAT is to induce musical imagery throughout the imagery component of the trial, this later group was classified as using an alternative Strategy; with only minimal musical imagery induced.

In addition, all participants who reached above Level 4 were asked if they possessed absolute pitch (AP), of which two self-reported they did. One reported that although they possessed AP they were not labelling the notes, just imagining the sound in their mind; hence they were categorised as using a musical imagery strategy. The other used an unusual alternative visual-motor strategy. This later individual was the only participant to reach above level 4 on the PIAT without the use of a musical imagery strategy. This participant was excluded from further analysis of strategy use and is considered in more detail in the discussion section. As it could be argued that AP possessors are likely to have clear long term mental categories which are highly likely to influence performance on the task, the following analyses were later re-run excluding both AP possessors, but it had no effect on any of the current results; as such the AP possessor who reported using musical imagery remains included in the results below.

Musical imagery strategy users were significantly more accurate on both Imagery (*t*(33) = 4.46, *p* < .001, *d* = 1.54) and Perception (*t*(33) = 4.35, *p* < .001, *d* = 1.50) trials. Significantly faster mean hit reaction times were found for Musical imagery strategy users in the Perception condition (*t*(33) = 2.62, *p* = .013, *d* = 0.90), though not the Imagery or Mental Arithmetic condition.

While musicians did not differ significantly from non-musicians in strategy use (*t*(33) = 1.61, *p* = .117, *d* = 0.56), there was a significant correlation between the MEI and strategy used (*r* = .55, *p* < .001), such that those with greater musical experience over their lifetime were more likely to report using a musical imagery strategy.

Finally, an independent t-test revealed that musical imagery strategy users reported significantly higher BAIS-C (musical imagery: M = 5.75, SD = 0.86; alternative strategy: M = 4.82, SD = 0.76; *t*(33) = 3.28, *p* = .002, *d* = 1.13), though there was no significant difference on BAIS-V (musical imagery: M = 5.38, SD = 1.08; alternative strategy: M = 4.86, SD = 0.66; *t*(33) = -1.61, *p* = .12, *d* = 0.56). They also had significantly higher debrief vividness scores (musical imagery: M = 3.93, SD = 0.84; alternative strategy: M = 2.96, SD = 0.75; *t*(33) = 3.47, *p* = .001, *d* = 1.20).

### Additional Imagery Performance Measures

The maximum level reached in the PIAT corresponds to the number of tones imagined per trial prior to the test probe. Both BAIS-V (*r* = .46, *p* = .005) and BAIS-C (*r* = .44, *p* = .007) were significantly correlated to maximum level attained. Musicians attained a significantly higher maximum level of performance than non-musicians (musicians: M = 4.04, SD = 1.00; non-musicians: M = 3.33, SD = 0.89; *t*(34) = 2.08, *p* = .045, *d* = 0.73). Maximum level was also correlated significantly with the MEI (*r* = .40, *p* = .015). Musical imagery strategy users reached a significantly higher level on the PIAT than alternative strategy users (musical imagery: M = 4.79, SD = 0.98; alternative strategy: M = 3.45, SD = 0.34; *t*(33) = 5.781, *p* < .001, *d* = 1.69). Maximum level reached is a more useful measure of imagery accuracy than Imagery percent correct, which does not account for the variability of difficulty in the levels of the task.

To capture how accurately participants progressed up through the levels (i.e. whether repeated mistakes caused them to drop a level, or whether they progressed up swiftly through to Level 5 and remained there), ‘Rate of Progression’ was calculated as the slope of the line of best fit of the level number over the 90 Imagery trials, (setting the intercept at trial 1 as Level 1). An independent t-test between the musicians and non-musicians revealed no significant difference in this Rate of Progression (musicians: M = 0.04, SD = 0.02; non-musicians: M = 0.03, SD = 0.02; *t*(34) = 1.92, *p* = .06). However there was a significant correlation with MEI (*r* = .44, *p* = .008). Musical imagery strategy users progressed significantly faster than alternative strategy users (*t*(33) = 4.56, *p* < .001). Rate of Progression also correlated significantly with both BAIS-V (*r* = .45, *p* = .006) and BAIS-V (*r* = .43, *p* = .009).

**Fig 3:**
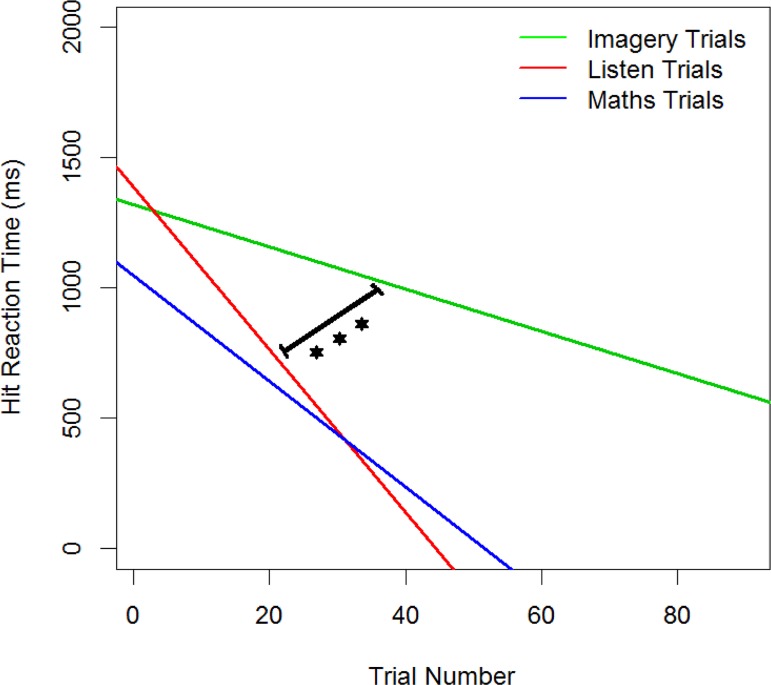
Rate of change of Reaction Time over trials.

The rate of change in reaction time as the participant moved through stages and levels of a condition provides an index of how quickly participants improved and is also an indication of the relative difficulty of the three conditions. ANOVA confirmed significant overall differences in difficulty (*F*(2,105) = 6.24, *p* = .003, η^2^ = .11). Post hoc paired t-tests showed that Imagery (M = -8.11, SD = 6.10) was significantly more difficult than Perception (M = -31.27, SD = 28.12): (*t*(35) = 5.62, *p* < .001, *d* = 0.94), but there was no significant difference between the Mental Arithmetic condition (M = -20.32, SD = 38.67) and the other two conditions (See Fig. 3).

### Multiple Regression Analysis

Regression analyses were conducted to evaluate which variables best predicted accurate performance on the PIAT, taking maximum level reached as the criterion variable. Eleven predictor variables (overall accuracies for Perception and Maths conditions; reaction times for Imagery, Perception, Maths Conditions; Musician [Y = 1, N = 0]; MEI; Strategy Use [Musical Imagery = 1; Alternative Strategy = 0]; BAIS-V; BAIS-C; Debrief Vividness) were included in an initial model and stepwise regression reduced the model to the minimal adequate hierarchical linear model, with only significant predictors. This resulted in a final model containing strategy use and BAIS-V, which significantly predicted the maximum level reached (*F*(2, 32) = 17.69, *p <* .001), and accounted for 53% of the variance in the maximum level score (R^2^ = .525; R^2^_adj_ = .495). The coefficients from this model are outlined in Table 4, under Model 2.

**Table 4:** Summary of multiple regression analysis for variables predicting Maximum Level Reached (N = 35)

	Model 1			Model 2			Model 3			Model 4		
Variable	B	SE B	β	B	SE B	β	B	SE B	β	B	SE B	β
Strategy Use	1.341	0.273	0.642** *	1.155	0.261	0.553** *	1.131	0.27	.552***	1.044	0.308	.50**
BAIS Vividness				0.356	0.135	.325*	0.336	0.144	.307*	0.346	0.137	.315*
Musician [Y = 1,N = 0]							0.133	0.287	0.061			
Musical Experience Index										0.474	0.676	0.1
R^2^_Adj_; R^2^; Δ R^2^	.405; .422; .422			.495. 525; .103			.483; .528; .003			.487; .533; .008		
F for change in R^2^	F[1,33] = 24.11***			F[2,32] = 6.935*			F[3,31] = 0.215			F[3,31] = 0.492		

Significance is denoted as

* = *p* < .05

** = *p* < .01

*** = *p* < .001.

A linear regression calculated the variance attributable to strategy use alone, given the high correlation between strategy use and maximum level (see Table 3). The result was significant (*F*(1, 33) = 24.11, *p <* .001), with strategy use alone accounting for 42% of the variance in maximum level (R^2^ = .422; R^2^_adj_ = .405). An ANOVA revealed that Model 2 (Strategy Use + BAIS-V) was significantly better than Model 1 (Strategy Use alone) (*F*(2, 32) = 6.935, *p* = .013), as seen Table 4.

Additional linear regressions assessed the effect of adding in musical training. Model 3 and 4 on Table 4 show the addition of musician category group and MEI respectively. Neither model was a significant improvement, with R^2^ increasing from Model 2 by only .003 and .008 respectively. This suggests that maximum level reached is either not strongly predicted from musical experience (only from Strategy Use and BAIS-V), or that the influence of strategy use or BAIS-V are mediating the relationship between performance of the PIAT and musical training.

To test these possibilities, mediation analysis was run using MEI as the predictor, strategy use as the mediating variable and maximum level reached as the outcome variable. Logistic regression confirmed MEI significantly predicts strategy use (*z* = 2.817, *p* = .005), and linear regression confirmed strategy use significantly predicts maximum level (*β* = 0.64, *p* < .001). The direct effect of MEI predicting maximum level went from significant (*β* = 0.45, *p* = .006) to non-significant when controlling for strategy use (*β* = 0.14, *p* = .37), suggesting the mediation was substantial. This result suggests that musical training, though related to the performance on the PIAT (as measured by the maximum level reached), is only predictive of performance due to the impact it has on strategy use. MEI did not significantly predict BAIS-V (*β* = 0.24, *p* = .171), and so it can be ruled out as a mediating factor in the relationship between musical training and performance.

**Fig 4:**
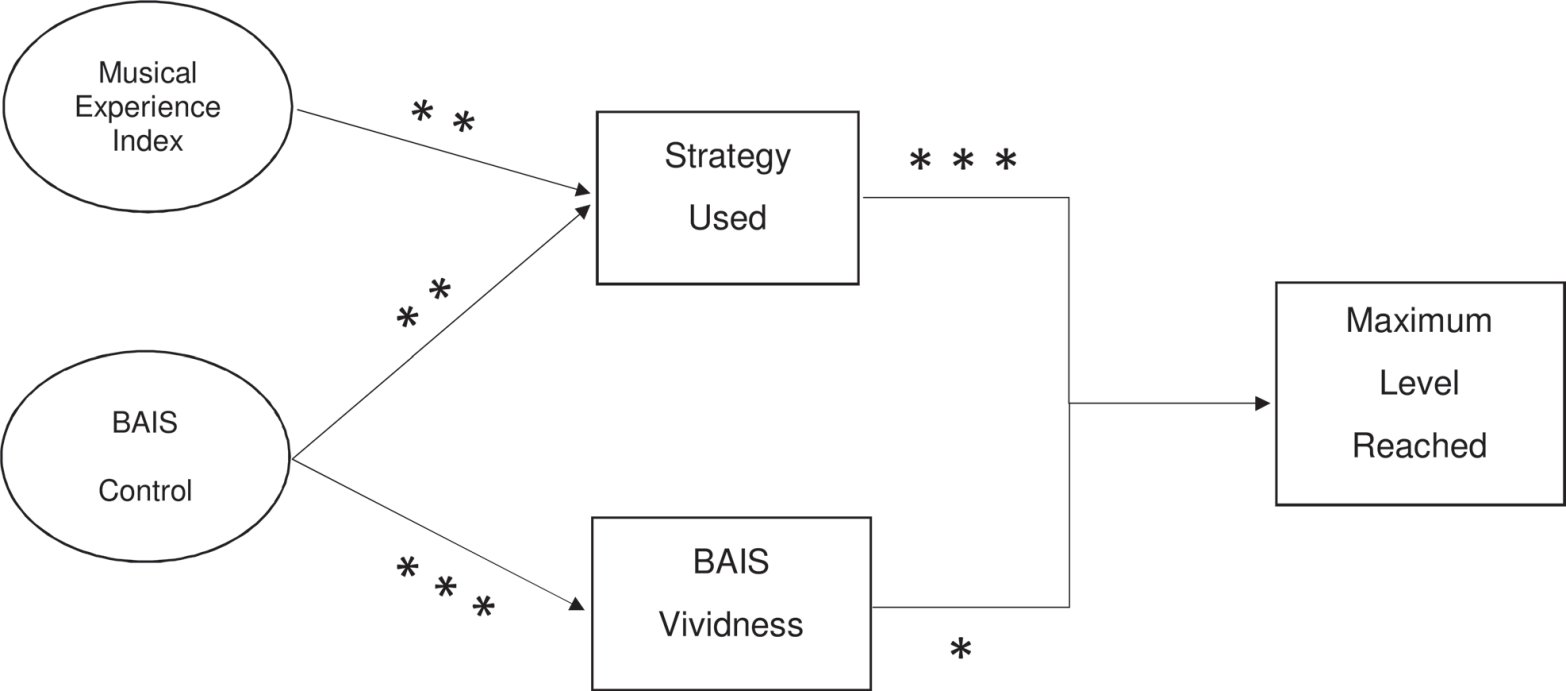
Regression model of maximum level of performance on the PIAT.

Finally, the role of BAIS-C was investigated to see how it fits into this model of prediction. Logistic regression showed that BAIS-C significantly predicted strategy use (*z* = 2.635, *p* = .008). The direct effect of BAIS-C predicting maximum level went from significant (*β* = 0.44, *p* = .007) to non-significant when controlling for strategy use (*β* = 0.20, *p* = .19), again suggesting that strategy use was substantially mediating the relationship between BAIS-C and maximum level reached. Linear regression also confirmed BAIS-C significantly predicted BAIS-V (*β* = 0.71, *p* < .001), but when controlling for BAIS-V, both variables were no longer significant in predicting maximum level reached. Fig. 4 describes the final model for maximum level reached showing strategy use and BAIS-V as the main predictors, and MEI and BAIS-C separately predicting strategy use (though not when combined), and BAIS-C also predicting BAIS-V.

**Fig 5:**
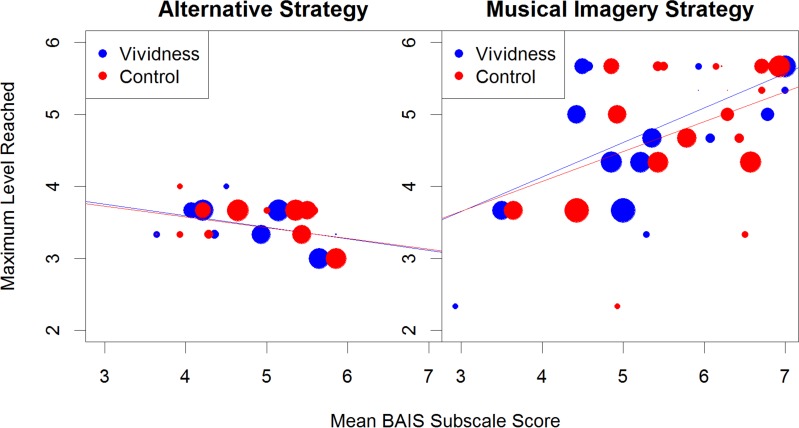
Relation between BAIS scores and maximum level of performance for the different strategy groups. Larger circles indicate greater musical experience.

In summary, musicians were significantly more accurate than non-musicians for Imagery but not Perception trials, and there was no significant group difference in reaction times. BAIS-C scores were positively correlated with performance on both Imagery and Perception trials, as well as other measures such as strategy used and MEI. In contrast BAIS-V was positively correlated with Imagery accuracy and debrief vividness. Regression analysis showed that the factors that contributed most to better performance on the PIAT were strategy use and BAIS-V. Participants with more musical experience were more likely to use a musical imagery strategy, and therefore perform better at the task. BAIS-C also predicted both strategy use and BAIS-V. Fig. 5 summarises the relationship between the main variables graphically and shows the differences in maximum level reached and both BAIS subscale scores, between the two strategy use categories. The size of the point on the graph is in proportion to the MEI, such that the larger points indicate a greater amount of life years spent participating in musical activity. It is interesting to note from Fig. 5 that within the subset of 21 participants who used a musical imagery strategy, musical experience (MEI) did not predict the maximum level attained (*r* = .14, *p* = .536). However there is a significant relationship between BAIS-V and maximum level attained (*r* = .53, *p* = .013), though not with BAIS-C and maximum level attained (*r* = .36, *p* = .105).

## Discussion

The present results confirm the effectiveness of the PIAT for inducing musical imagery, and provide insights into the roles that musical training, auditory vividness and mental control play in successful imagery performance. The PIAT was highly effective in inducing mental imagery of musical pitch in participants, in that successful performance on the task was highly dependent on the use of a musical imagery strategy, rather than an alternative strategy that used minimal musical imagery. Participants using an alternative cognitive strategy were (bar 1) unsuccessful at reaching above Level 4 on the Imagery task, with musical imagery strategy users significantly outperforming alternative strategy users on all measures of accuracy. The exception, a participant with 10 years of piano training reported, reached level 5 using a visual-motor imagery strategy. This individual reported visualising the keys on a piano and playing them with their hand. However, this individual also possessed AP and knew which pitches and scale degrees were being played. As the only individual to adopt a visual-motor imagery strategy, this participant was considered individually and excluded from the statistical analysis of strategy use and multiple regression analyses. The participant achieved 98% accuracy for the Imagery condition, and was below the sample’s mean for BAIS scores on both subscales. The other participant who also self-reported having AP, reported using pitch imagery. This participant was within one standard deviation of the mean for MEI, BAIS-V and BAIS-C among musical imagery strategy users, and her exclusion from the analyses did not significantly alter any of the findings (all *p* values remained within the stated significance level). Hence, this second AP participant was included in the analyses.

In comparison to previous musical imagery tasks, the present procedure has several advantages. First, it provides a number of objective and complementary behavioural measures of accuracy (percent correct per condition, maximum level attained, rate of progression through the levels) and indices of reaction time (mean hit reaction time and rate of change of reaction time per condition). These behavioural measures revealed that better performance on the PIAT was associated with the use of a musical imagery strategy. The measures also showed musicians were only significantly more accurate on the Imagery but not on Perception trials, and were not significantly different in reaction times. This result is consistent with the findings of Aleman et al. [26], who had participants mentally compare pitches of notes corresponding to lyrics taken from familiar songs. The pattern of results also suggests the PIAT is not biased towards musicians, unlike the maintenance paradigm used by Kuchenbuch et al. [27], in which non-musicians are significantly worse than musicians on perception trials, and were at chance level for the imagery trials.

The second advantage of the PIAT is that it requires participants to actively manipulate a pitch image, rather than just maintain it. This type of manipulative pitch imagery investigation has only up until now been done with more difficult tasks such as mental reversal of melodies or simpler pitch transposition of melodies [2, 5, 7].

Thirdly, unlike previous protocols for inducing imagery, random sequences were manipulated in the imagery component of the trial. This design confers variety and flexibility to help minimise confounds, such as a familiarity with a melody / or familiarity with a probe combination that could be learnt over a task. For example Level 1, Stage 1 (with number of initial arrows / tones set to 3, starting note of tonic, in the key of C Major, and with only 1 imagined arrow) had 14 different possible combinations. This number increased dramatically as the participants move through the levels and stages of the task. Not only did the pitch sequence vary randomly, but the length of the initial set-up sequence varied randomly, so that participants unaware when the imagery component of the trial would begin.

Fourthly, the staircase design allowed participants to progress through levels at their own rate, while at the same time accommodating individuals with a wide range of musical experience. While 4 non-musicians did fail to progress past Level 1, 3 non-musicians made up the group of 18 who progressed past Level 4 on the PIAT. One of these non-musicians also had a MEI of 0 indicating no musical participation at all. As expected, musicians performed better on the task, with 96% of them getting to Level 3 or above. Nonetheless, 63% of the non-musicians were also able to attain a maximum level of 3 or above, confirming that the PIAT can be used to induce pitch imagery in both musicians and non-musicians.

Finally, the basic design of the PIAT allows for very comparable control conditions which do not require the use of musical imagery. In the perception condition, the participants were only made aware when the probe screen appeared that the trial did not require imagery, and therefore was a Perception trial. This ensured participants were actively listening throughout the trial, in anticipation that imagery may be required at any point. It also provided an identical probe presentation for direct comparison to the Imagery trials. Importantly, the mean reaction times for Imagery and Perception trials were not significantly different (even though accuracy was higher in Perception trials), due to the use of identical probes in the two conditions.

Mental arithmetic provided a second control condition that required no musical imagery, but did require increasing mental capacity as the levels increased and the calculations became longer. The difficulty of the mental arithmetic trials were comparable to the imagery trials, given the similar accuracy measure scores, and decrease in reaction time over the task. These results suggest participants learned the two tasks at a similar rate. Interestingly, musicians performed more poorly than non-musicians on mental arithmetic trials, but the reasons for these differences are unclear. Musical imagery strategy users were generally faster than alternative strategy users (as seen in the negative correlations between Reaction Times and Strategy Use in Table 3), and musicians using an alternative strategy (N = 7), appear to be responsible for the overall slower reaction times by musicians.

A second aim was to investigate the relative importance of musical training, imagery vividness and mental control in musical imagery. Regression analysis showed that musical experience did not contribute significantly to a linear model of prediction for maximum level reached in the PIAT. Further mediation analysis showed that the relationship between MEI and maximum level reached in the PIAT was substantially mediated by strategy used; such that more musical activity over the lifetime increased the likelihood of a musical imagery strategy being used in the PIAT. It was the use of such a strategy that lead to better performance, rather than simply musical experience. These results, though surprising, may be due to the nature of the imagery task. Studies have shown in the visual domain that object imagery (maintenance) and spatial imagery (manipulation) had differing behavioural and psychometrical properties with visual artists excelling at object imagery and scientists excelling at spatial imagery [15]. A similar discrepancy in the auditory domain may be seen in expertise among musicians and non-musicians, with musicians performing better at maintenance than manipulation of musical images.

Both BAIS subscales, though highly correlated to each other, were significantly correlated with different variables; confirming they index at least partially different aspects of the auditory imagery experience. BAIS-V correlated with the vividness rating participants gave after completing the PIAT (debrief vividness), suggesting that this more abstract auditory scale is associated with the subjective experience of musical imagery vividness during the PIAT. However it was the BAIS-C that correlated most significantly with Imagery and particularly Perception performance, suggesting that being able to manipulate sound images at will may be assisting with the anticipation of the perception of them. Pfordresher & Halpern [20] also showed a significant relationship between a perception task involving judgement about the relative height of two tones, and BAIS-C. However this is the first study to show a significant correlation between imagery performance and BAIS-C, presumably because the PIAT involves manipulation or change of the pitch image, which requires greater mental control than maintenance paradigms. The three main variables of interest (MEI, BAIS-V and BAIS-C) all correlated with the various accuracy measures of the PIAT. However only the BAIS-C was significantly correlated with imagery reaction times, indicating this measure is most strongly associated with overall imagery performance in this task.

A more complete picture of the relationships between these variables emerged through regression analysis, with strategy use proving to be the biggest single predictor of maximum level reached on the PIAT. Fig. 5 shows graphically that within the musical imagery strategy users, MEI is not as big a predictor of maximum level reached; with some participants with very little musical experience able to attain a higher level than others with considerable musical experience, but with lower BAIS-V and BAIS-C scores. The combination of strategy use and BAIS-V accounted for 53% of the variance within the maximum level reached. Although MEI and BAIS-C individually predicted strategy use, and BAIS-C predicted BAIS-V, their addition to the regression model was not a significant improvement. Hence BAIS-V is more important to predicting performance on the PIAT than MEI or BAIS-C.

Therefore while BAIS scores have been shown to be more important than musical experience in performance on this musical imagery task, it is clear the most important factor is the use of a musical imagery strategy. Interestingly, participants who reported using a musical imagery strategy were more accurate and had faster reaction times for Perception trials also, indicating that even when no manipulation of an auditory image was required, performance was facilitated by a musical imagery strategy.

It could be argued that the use of up and down arrows in the PIAT reflects a spatial conception of pitch that may encourage the use of a spatial imagery strategy to complete the task. However, arrows were presented merely to indicate which pitch to imagine next, and participants were explicitly instructed to imagine the sounds of the pitches. Indeed, any learned association with pitch height could have been used to guide imagery. We asked participants to describe the strategies they used in completing the musical imagery trials, and the most common and successful of strategy was musical imagery rather than visual imagery. More generally, the PIAT can be readily adapted to other culture-specific schemata. For example, the major scale reflects a western conceptualization of pitch, but the PIAT can easily be modified to alternate musical scales (e.g., pentatonic, slendro, whole tone).

## Conclusions

In this investigation, the PIAT was introduced as a powerful new protocol for assessing musical imagery. We confirmed that the PIAT reliably induces pitch imagery in individuals with a range of musical experience, particularly above Level 4. It entails the active manipulation of an auditory image that most non-expert musicians can readily perform. Our results showed competent performance on the PIAT requires active musical imagery and is very difficult to achieve using alternative cognitive strategies.

The PIAT provides a platform in which to address questions of individual differences in musical expertise in imagery performance, as well as the role of auditory vividness and mental control. More musical training, increased self-reported BAIS-V and BAIS-C were associated with better performance on the PIAT. Both BAIS subscales were important, as success in the task required more than the ability to just hear an image in the mind, but involved the ability to successfully manipulate or change that musical image. Our results also support our second hypothesis that both auditory imagery vividness and the ability to control auditory images are more important than musical training in contributing to success in this type of imagery.

The task is readily adaptable to neuroimaging studies of the neural correlates of pitch imagery. The basic protocol also lends itself to investigations of aspects of musical imagery including loudness, tempo or rhythm. For example, a future rhythm imagery task could involve the presentation of a simple rhythmical pattern and arrows pointing either upwards/downwards to increase/decrease divisions in the beats or left/right to either mentally reverse or maintain the simple pattern. A future study could compare imagery performance on different types of pitch scales; though it is expected that both musicians and non-musicians would perform poorly when unfamiliar scales are used, and if the task is too difficult it may encourage the use of alternative strategies.

Feedback in the PIAT was included to facilitate acquisition of task performance. It could be argued that feedback could have influenced ratings of the vividness of musical imagery, in that participants might assign lower ratings of vividness should their overall imagery performance have been perceived as poor. We acknowledge this possibility but maintain that the benefits of feedback (at least during initial learning of the task) outweigh the potential disadvantages. More explicit instructions of the types of musical imagery strategies that should be used, as well as the alternatives strategies that should be consciously avoided, may also lead to a higher percentage of participants adopting the desired auditory imagery strategy.

Looking forward, the PIAT can also be used to address other theoretical issues surrounding musical imagery. First, unlike the visual domain, where it has been demonstrated that primary visual cortex is employed during visual imagery [28], there is debate concerning whether primary auditory cortex is involved in musical imagery [1, 12]. Second, it is unclear how mechanisms underlying musical imagery can be integrated into current models of auditory memory [29, 30]. Baddeley suggests auditory imagery may reside in the “phonological loop” which includes both an auditory memory store and an articulatory rehearsal process, rather than the “central executive” [31]. Musical imagery also has practical implications. There is considerable interest in the use of imagery and mental practice in music education, and in successful group performance [32, 33]. Finally, a clearer understanding of the links between musical imagery and perception may prove beneficial for patients with hearing loss, or for post-lingual recipients of cochlear implants; who may have functional musical imagery capabilities but have reduced capacity to perceive music. For example, incorporating the PIAT in music-based training for these patients may be beneficial, particularly for individuals with higher BAIS-C scores, given its association with Perception performance.
